# Health Beneficial Effects of Moomiaii in Traditional Medicine

**DOI:** 10.31661/gmj.v9i0.1743

**Published:** 2020-08-27

**Authors:** Solmaz Rahmani Barouji, Amir Saber, Mohammadali Torbati, Seyyed Mohammad Bagher Fazljou, Ahmad Yari Khosroushahi

**Affiliations:** ^1^Department of Persian Medicine, School of Traditional Medicine, Tabriz University of Medical Sciences, Tabriz, Iran; ^2^Student Research Committee, Tabriz University of Medical Sciences, Tabriz, Iran; ^3^Department of Nutritional Sciences, School of Nutritional Sciences and Food Technology, Kermanshah University of Medical Sciences, Kermanshah, Iran; ^4^Department of Food Science and Technology, Faculty of Nutrition and Food Sciences, Tabriz University of Medical Sciences, Tabriz, Iran; ^5^Drug Applied Research Center, Tabriz University of Medical Sciences, Tabriz, Iran; ^6^Department of Medical Nanotechnology, Faculty of Advanced Medical Science, Tabriz University of Medical Sciences, Tabriz, Iran

**Keywords:** Persian Medicine, Humic Acid, Fulvic Acid, Immunomodulation, Antidiabetic, Antineoplastic

## Abstract

Traditional medicine (TM) that developed over the years within various societies consists of medical experimental knowledge and practices, which apply natural methods and compounds for general wellness and healing. Moomiaii as a pale-brown to blackish-brown natural exudate is one of the natural compounds in traditional medicine that has been used over 3000 years in many countries of the world especially in India, China, Russia, Iran, Mongolia, Kazakhstan and Kirgizstan. We reviewed all English-language studies about Moomiaii that we accessed them. In traditional medicine, many beneficial activities have been attributed to Moomiaii and to its main constituents, Humic acid and Fulvic acid, which are widely used to prevent and treatment of different diseases. Some modern scientific investigations showed that Moomiaii as a safe dietary supplement can be beneficial in various health complications. Even though the beneficial effects of Moomiaii have been confirmed in traditional and modern medicine, it seems that additional *in-vitro/in-vivo* studies and comprehensive clinical trials are necessary to explain the whole mechanisms of action and to determine the effective doses in various diseases. We discuss and clarify the claimed health beneficial effects of Moomiaii in some wide-spread diseases regarding its anti-ulcerogenic, immunomodulatory, antidiabetic, antioxidative and anticancer properties.

## Introduction

**Figure 1:**
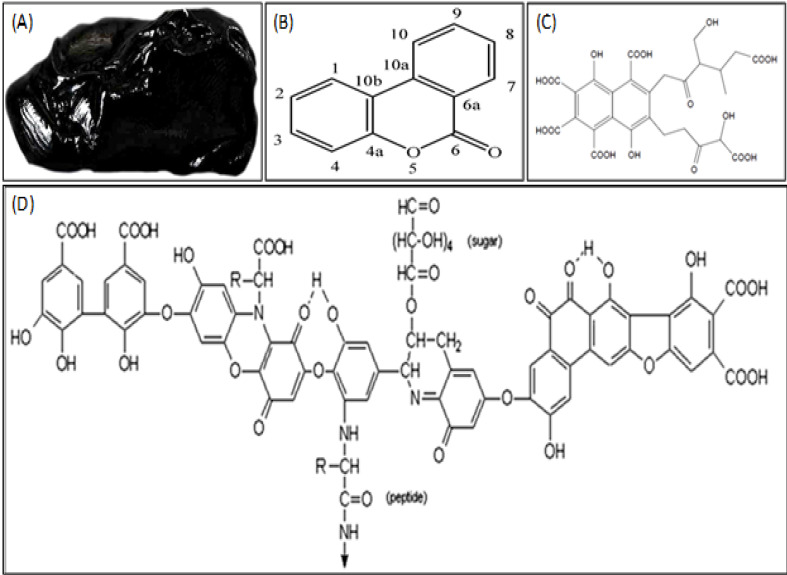
**(A):** piece of Moomiaii known as a sticky, brown to the blackish natural substance, **(B): **dibenzo-α-pyrones, **(C): **Fulvic acid, and **(D): **Humic acid.

Traditional medicine (TM) refers to the knowledge, skills, and practices based on the theories, beliefs, and experiences indigenous to different cultures, used in the maintenance of health and in the prevention, diagnosis, improvement or treatment of physical and mental illness [1]. In this way, various studies are being performed about TM and some health beneficial effects of natural medicinal compounds in several diseases have been proved [2]. The TM was divided into different systems, including traditional Persian medicine (TPM), traditional Arabic medicine, traditional Chinese medicine (TCM) and traditional Indian medicine (Ayurveda) [3]. In this regard, the Moomiaii is a unique and renowned compound that commonly used in TM systems. This compound, also known as Shilajit, Silajita, Marathi or Gujarati (in Hindi), Asphalt (in English), Silajatu (in Bengali), Rock juice (in Tibet), Conqueror of mountains (in Sanskrit), Hajarul-Musa or Araq-al-jibal (in Arabic), Moomiaii or Mumnaei (in Persian), μούμια (in Greek), Myemu (in Russian), Mumie (in German), Mineral Pitch, Jew’s Pitch, Mineral Wax, and Brag-shun, as a pale-brown to blackish-brown natural substance has been used over 3000 years as a rejuvenator, adaptogen compound (Figure-1 A) [4]. The Moomiaii is formed in very small quantities in some specific weather conditions and obtained from steep rocks at high altitudes when it becoming less viscous and extruded from the layers of these rocks during summertime [5]. In some parts of the world, the Moomiaii is mostly flowed out from the layers of the rocks such as the Himalayan mountain range, Iran, Afghanistan, Nepal, Bhutan, Pakistan, China, Tibet, Caucasus and other countries like Russia, Tibet, and Norway [6]. In Iran, the Moomiaii flowed out mostly from the Darab mountains of the Fars province and the mountains of Estahban and Kohgiluyeh [3]. Razi, as a famous Persian physician, has prescribed the Moomiaii in different situations and diseases like trauma, pain relief, fractures, injuries, headaches, tonic, brain tonic, earache, asthma, spleen pain, and pediatric seizures [7]. Based on different theories about Moomiaii’s source, this compound generated based on three main biological, geological and bio-mineralogical theories. The biological theory suggested that Moomiaii produced from dead plant residues or animal stools in some physiochemical conditions. The geological theory suggested that Moomiaii is a product of long geological processes. Also, the bio-mineralogical idea proposed that this compound is a by-product of mechanical contamination of liquefied Moomiaii precursor and mineral components [8]. Since the Moomiaii mostly finds in the sedimentary rocks, other opinions suggested that this compound is mostly made from marine invertebrate animals like mollusks/ammonites [9]. Different factors including the region of the production, plant-species, the geological nature of the rock and soil, local temperature, humidity, and altitude, etc. affect the composition and therapeutic properties of Moomiaii [10]. These affective factors influence the composition and the ratio of components in the Moomiaii despite the similar physical characteristics in different areas of the world. Generally, the Moomiaii is composed of organic (60–80%) and inorganic (20–40%) compounds and trace elements (Fe, Ca, Cu, Zn, Mg, Mn, Mo, P) [11]. Moreover, the Moomiaii is soluble in the water, and about 30-50% of its ingredients moved into the liquid phase and depending on the purity of the sample the quantity of sediments is different [12]. Also, based on molecular weight assessment, there are three main chemical components in the natural mixture of Moomiaii, including; 1) low and medium molecular weight compounds containing free and conjugated dibenzo-pyrones (e.g. fatty acyl, aminoacyl, lipoidal). 2) high molecular weight dibenzo-pyrones-chromoproteins (DCPs), containing trace metal ions and coloring materials such as carotenoids and indigoids and 3) Metallo-humates like fulvic acids and fusims with dibenzo-pyrones in their core nuclei [9].

However, the results of different examinations about detection of precise compound(s) of Moomiaii showed that 60–80% of its compound is made of humus and the rest comprised of benzoic acid, hippuric acid, fatty acid, ichthyol, ellagic acid, resin, waxy materials, gums, albuminoids, vegetable matter, triterpenes, sterol, aromatic carboxylic acid, 3,4-benzocoumarins, amino acids, and phenolic lipids [13]. As well, it seems that dibenzo-α-pyrones (Figure-1 B), Fulvic acid (FA) (Figure-1 C) and Humic acid (HA) (Figure-1 D) as the most important and active compounds of Moomiaii are responsible for various health beneficial effects of it like improvement of memory and neuroprotective, anti-inflammatory, and antioxidant activities [14]. Also, these effective compounds are able to act as carrier molecules for other active compounds [15]. Moomiaii was prescribed in different doses for various health problems such as genitourinary disorder, jaundice, gallstone, gastrointestinal disorders, enlarged spleen, epilepsy, hypersensitivity, nervous disorder, chronic bronchitis, tuberculosis, eczema, anemia, and diabetes [16]. The fungal contamination and particularly mycotoxins are the most important factor that limits the general prescription of Moomiaii in the world [17]. In addition, the traditional medicine specialists claim that Moomiaii is effective in the lack of sexual desire, treating kidney stones, bone pains, and fractures, osteoarthritis, spondylitis, edema, piles, aging, rejuvenation, internal antiseptic, adiposity, anorexia, and fat reduction [10]. According to anti-inflammatory, anti-oxidative, anti-mutagenic and immuno-modulatory properties of FA and HA, some evidence suggested that Moomiaii can be a potential cancer-preventive agent [11]. In this way, numerous studies have been performed to examine and prove these beneficial effects. Bhattacharya and Sen showed that Moomiaii possesses antiradical-antioxidant activities which confirmed the therapeutic effects of Moomiaii as an Ayurvedic rusayun against oxidative stress-induced diseases and geriatric complaints [18]. Additionally, different doses of Moomiaii reduced the blood glucose levels and also had favorable effects on the lipid profile in rats [19]. As well, Moomiaii extract enhanced the synthesis of nucleic acids and increased the transportation of minerals into muscle and bone tissues [12]. Regarding various investigations and claims about therapeutic properties of Moomiaii in TM and Ayurveda and Siddha systems of medicine and lack of comprehensive evaluations in this issue, this review article aims to collect and summarize the performed investigations about health beneficial and therapeutic effects of Moomiaii which using in TM in different parts of the world.

## Search Strategy

 PubMed, Medline, Google Scholar, Scopus, SID, Embase, IranMedex and IRANDOC as main scientific databases, were searched for articles in this field without limitation about language from 1965 to 2018 with different keywords such as; traditional Persian medicine (TPM), traditional Indian medicine (Ayurveda), traditional Chinese medicine (TCM), and traditional Arabic medicine, Moomiaii, Mumnaei, Mumijo, Shilajit, Silajita, Marathi, Gujarati, Asphalt, Silajatu, Rock juice, Conqueror of mountains, Hajarul-Musa, Araq-al-jibal, Myemu, Mumie, Mineral Pitch, Jew’s Pitch, Mineral Wax, and Brag-shun.

###  Anti-ulcerogenic Effects of Moomiaii

Gastric ulcer is one of the most common illnesses of the digestive tract with significant rates of morbidity and mortality worldwide [20]. The prevalence of this illness in the western population is about 2.4% and the annual incidence rate is 0.10% to 0.19% [21]. In China, the prevalence of gastric ulcers is very high (6.07% in the general population), and approximately 22.5% of people with gastrointestinal symptoms have a gastric ulcer [22]. The damage through increasing gastric acid secretion may extend into the muscularis propria layer of the gastric epithelium and make more detrimental effects [23] involved in the etiology of gastroduodenal ulcers including acid-pepsin secretion, mucosal barrier, mucus secretion, blood flow, cellular regeneration, endogenous protective agents [24]. Since inflammation and oxidative stress are the main causes of the incidence of gastric ulcer, Moomiaii may affect through its anti-inflammatory and antioxidant activities [25]. It seems that some active metabolites presence in Moomiaii can attenuate acid-pepsin secretion, cell shedding, and gastric ulcer index [24]. Moreover, various studies revealed that dibenzo-α-pyrones and FA are responsible for the antioxidant effects of Moomiaii [26]. These active compounds can increase the activities of several key enzymes in the oxidative pathway such as superoxide dismutase (SOD), catalase (CAT) and glutathione peroxidase (CPX) [27]. Based on the previous investigation results, the treatment with Moomiaii extracts significantly protects against an acetic acid-induced ulcer in the rat model and showed regenerative and repairing activities in histopathological assessments suggesting that Moomiaii has potent anti-ulcer activity and this property probably performs through the reduction of gastric acid secretion and pepsin levels [24]. Based on the findings of available studies the anti-ulcerogenic activity of Moomiaii is attributed to its antihistamine, anti-serotonin, anti-oxidative, anti-inflammatory and free radicals scavenging effects [28] through compounds with strong anti-inflammatory properties like benzoic acid (up to 7–8%), fulvic acids, 4’-methoxy-6-carbomethoxybiphenyl, and tirucallane-type triterpenoids [29]. Some investigations suggested that the metabolism of arachidonic acid, platelets, macrophages, and smooth muscle cells are involved in the production of reactive oxygen species which lead to gastric mucosal damage. Thus, Moomiaii, as an antioxidant agent, inhibits lipid peroxidation and free radical-mediated process protects the gastric mucosa from oxidative damage and accelerates the healing of gastric ulcers or its anti-stress activities have significant effects on the healing of gastric ulcer disease [30].

###  Immunomodulatory Effects of Moomiaii

Moomiaii immunomodulatory activities have been proved by some *in-vitro* and *in-vivo* investigations. The effects of Moomiaii on peritoneal macrophages and cohabiting fibroblasts in mice has shown the time-dependently increase of the white blood cells (WBC) activity and significantly increase the amount of heteromorphic and phagocytic cells after treatment compared to the placebo [31]. The findings of the immunomodulatory effects of Mumijo suggested that Mumijo improves the lytic potential of polymorphonuclear leukocytes and administration of 200–600 mg/dose of Mumijo in mice, made significant morphological and phagocytotic alterations in peritoneal macrophages [32]. The results of the effects of 25 and 50 mg/kg i.p. of shilajit on the rat’s brain monoamines levels indicated a significant decrease in the level of 5-hydroxy tryptamine and 5-hydroxy indole acetic acid and an increase in the level of dopamine, noradrenaline and its metabolites in rat’s brain. The created alterations in the level of neurotransmitters are comparable with increased immune activity [33]. Besides, shilajit is able to lyse 51Cr labeled tumor cells via reinforcement of activated lymphocytes with lytic potential and making of T-cell mediated cytotoxicity. Some components of Moomiaii like FA, dibenzo-α-pyrones, and 3,8-dihydroxydibenzo-pyrones have immunoregulatory effects and can inhibit the proliferation of Ehrlich ascites tumor cells without significantly affecting the number of dead cells in doses of 400 g/mouse [34]. Furthermore, the main constituents of Shilajit stimulated peritoneal macrophages and activated splenocytes of tumor-bearing animals at early and later stages of tumor growth [12]. Findings in two experiments using FA- and HA-supplemented diets in rats for 26 days showed a dose-dependent increase of plasma TSH and a decrease in the T4/T3 ratio in rats. Also, both FA and HA stimulated the immune response without significant differences in the lymphocyte stimulation test suggestting that FA and HA supplementation resulted in strong humoral immune stimulation [35]. Furthermore, HA and FA have illustrated immunostimulatory [36], antimicrobial [37], anti-inflammatory [38], antiviral properties [39], anti-allergy [40] *in-vitro*/ *in-vivo*. In the anti-inflammatory phase, FA can decrease the secretion of proinflammatory mediators from cells [41]. Treatment with FA at a dose of 200 μg/mL decreased tumor necrosis factor-alpha (TNF-α) expression after exposure to the endotoxin Lipopolysaccharide (LPS) in differentiated human monocytes (U937) [42]. Also, FA and solubilized sludge (SS-FA) can reduce cyclooxygenase 2 (COX2), prostaglandin E2 (PGE2) secretion after homocysteine stimulation in primary human monocytes [43], and also B-hexosaminidase, histamine, TNF-α, interleukin-4 (IL-4), and IL-13 release in immunoglobulin-E-sensitized mast cells and basophil cells [44]. Moreover, in-vivo studies indicated that topically application of coal-derived FA (oxifulvic acid) in 23 healthy volunteers allergic to grass or house dust mite allergen reduced wheal and flares size after allergen challenge and inhibited stimulated inflammatory reaction similar to the application of hydrocortisone drug [45]. Also, treated sensitized mice with oxifulvic acid caused a significant reduction in ear swelling compared to steroid medication measurable toxicity [38]. Furthermore, oral administration and topical application of carbohydrate-derived fulvic acid (CHD-FA) inhibited carrageenan-induced inflammation paw edema in rats without symptoms of systemic toxicity and these effects were comparable to nonsteroidal anti-inflammatory drugs. Topically administration of CHD-FA showed the antimicrobial and anti-inflammatory properties by accelerating effects on the healing of methicillin-resistant *Staphylococcus aureus*-infected and multidrug-resistant *P. aeruginosa* wounds in animal models [40]. The hydrated bis-dibenzo-pyrone ferrate complex structures, the main bioactive compounds of Moomiaii, with part of the protected iron coordination site(s) from Fenton-Haber Weiss type reactions being exposed and interact with oxygen in the singlet state or systemic hydrogen peroxide would produce phagocytic agents that would oxidize/destroy the noxious particles. Moomiaii makes conformational alterations and morphological changes in the exposed cells and causes selective oxidoreductase activities through created transition states that encompass several bonds [46]. Additionally, biphenyl and benzocoumarin compounds of Moomiaii possess significant antiallergic activities in eczema and psoriasis disease (0.2 g/d, per os, two 10 day cycles with 5-day break) via mast cell stabilization and significant reduction of their degranulation [32].

###  Anti-diabetic Properties of Moomiaii

Diabetes mellitus (DM) which mostly known as diabetes, is a group of metabolic disorders with some classic symptoms including hyperglycemia, polyuria, polydipsia, and polyphagia [47]. The blood glucose increasing leads to the poor protein synthesis and other metabolic disorders such as acidosis [48]. Mumijo can decrease hyperglycemia and increase SOD activity in pancreatic β-cells in diabetic rats induced by the reduction in superoxide dismutase (SOD) activity in defected β-cells [19]. The effects of three different doses of shilajit alone and a combination with either glibenclamide or metformin for 4 weeks were assessed on blood glucose and lipid profile in euglycemic and alloxan-induced diabetic rats and the results showed a significant decrease of blood glucose levels with positive effects on lipid profiles [19]. The effects of subcutaneous administration of processed shilajit together with insulin on plasma glucose level in streptozotocin-induced diabetic rats demonstrated the reinforcement impact on the insulin-induced hypoglycemia [16]. The effects of shilajit (two capsules 500 mg each; Dabir India) on 61 diabetic subjects of either sex who were on a steady dose of glibenclamide for 30 days findings displayed a significant decrease in lipid peroxidation, malondialdehyde, the values of superoxide dismutase (SOD) and glutathione peroxidase level and also a significant increase in catalase values of diabetic subjects compared with their higher pretreatment values [49]. Also, long-term treatment with Mumijo showed pancreatotrophic action and increased the number of β-cells of the pancreas which lead to enhanced sensitivity of pancreatic-cells and high amounts of insulin secretion in response to hyperglycemia [19].

###  Antioxidative and Antineoplastic Activities of Moomiaii

Cancer is the second leading cause of death after cardiovascular disease and with approximately 7 million deaths every year worldwide. The main etiologic factors for initiation and progression of cancer are toxins, free radicals, mutagens, heavy metals, blood sugar, virus, radiations and many other factors including inflammation. According to some investigations, it seems that Moomiaii can play an important role in cancer chemoprevention and possibly in its treatment. Moomiaii and its main constitutes FA and HA, as a nutritive, non-toxic/natural and rejuvenating tonic compound without reported side effects possess several favorable activities in favor of anticancer effects such as anti-mutagenic, antitumor, antioxidant, antitoxic, anti-inflammatory, antiviral, heavy metal chelating, photo-protective, immunomodulatory properties [50]. The reactive oxygen species (ROS) can damage DNA and increased destructions on DNA via disrepair or imperfect repair may result in mutagenesis and cancerous transformation subsequently [51]. Besides, ROS has an important role in the promotion phase which is known by the induction of cell proliferation, apoptosis inhibition, and growth of initiated cells [52] by modulating some genes in oncogenesis pathways such as NFκB, Nrf2, HIF, and p53 [53]. Furthermore, oxidative stress can stimulate proliferative pathways like ERK/MEK and PI3K/AKT and inactivate pro-apoptotic proteins and upregulation of antiapoptotic genes [54] at the low levels of ROS. As well, the production of more quantities of ROS contributes to the progression phase of cancer through induction of the mutation, inhibition of antiprotease, upregulation of matrix metalloproteinases (MMPs) and promoting angiogenic response and metastasis [55]. Interruption in the precise balance between oxidants and antioxidants may be a critical factor in occurring of cancer, which leads to oxidative damage to normal cells and tissues [51]. The natural antioxidants such as Moomiaii and its main constituents, HA and FA, can be efficacious agents for the prevention of cancer by performing anti-lipid, per-oxidative effects against NO or OH and significant radical scavenging activities [11]. The processed shilajit indicates the antioxidant activities and is able to regenerate ascorbic acid and neutralize sulfite anion, hydroxy and nitric oxide free radicals by its dihydroxybenzo-α-pyrones, protect methyl methacrylate against hydroxyl radicals and inhibit the polymerization of methylmethacrylate by the sulfite-free radicals [10]. Shilajit has inhibited lipid peroxidation induced by cumene hydroperoxide and ADP/Fe++ complex and decreased the rate of oxidation of reduced glutathione in a dose-dependent manner in rat liver [31]. The ultraviolet (UV) waves develop mutations and carcinogenesis and caused long-term DNA damage by forming the thymine dimer in the DNA [50]. The HA reduces the penetration of the high-energy wavelength lights through its UVB-absorbing activity and can protect mountaineers from sunburn [56]. Various investigations have shown that some viral infections are responsible for the development of cancer including Hodgkin lymphoma, Burkitt’s lymphoma, cervical cancer which is related to immunosuppression and HIV, Human Papillomavirus (HPV) and Kaposi sarcoma herpesvirus (KSHV) respectively [57]. In HIV patients, Moomiaiis’ HA can reduce the replication of the virus and HIV-related infections [50], and also stimulate and increase the T-lymphocytes [58], IL-2 production by TH1 cells [59]. Also, Moomiaii alongside antiretroviral therapy in HIV-infected patients increases the CD4 level, ameliorates the general situation and improves vomiting, depression, appetite, nausea, diarrhea, weight loss, fever, and anemia [50]. Also, HA with its antiviral and cytotoxic properties can protect against Herpes simplex virus-1 (HSV-1) which is responsible for genital and oropharyngeal cancers through blocking the HSV replication. These beneficial functions are related to carboxyl together with the hydroxyl groups [60]. Moomiaiis’ FA can protect additionally against cancer and related cancer-causing viruses and as a dietary supplement is also effective on viral respiratory illnesses in children [61]. The HA can induce apoptosis via reduction of mitochondrial membrane potential (DΨm), cytochrome c release, activation of caspase-3, -8, and -9, degradation of poly ADP-ribose polymerase (PARP), dysregulation of Bcl-2 and Bax, and upregulation of p53 and phosphorylated p53 (p-p53). Likewise, the HA can stop the cell cycle progression in the G1 phase by decreasing cyclin D1, CDK4, cyclin E, CDK2, and hyperphosphorylated retinoblastoma protein (pRb) in a time-dependent manner [62]. On the other hand, the treatment with shilajit can prevent radiation-related ovarian damage by blocking the apoptotic pathways by decreasing the expression of key apoptotic genes like p53, Bax, and caspase-3 [63] in normal cells.

 This evidence from various studies suggested that Moomiaii and its main constituents as a dietary factor may reduce the risk of cancer initiation/progress and can inhibit tumor growth via different pathways such as free radicals scavenging, UV absorbing, anti-inflammatory, anti-viral activities, and anti-proliferative/pro-apoptotic properties.

## Discussion

Moomiaii was used for several years as a well-known natural remedy with several beneficial effects in different illnesses. Most of the medieval Persian physicians like Razes prescribed Moomiaii as healing water (Abdaroo) to the treatment of bone fracture and gastrointestinal diseases. Moreover, other Iranian physicians such as Hakim Momen (in the Tohfat-almomenin), Khajeh- Nassir-al-din-Toossi (in the Tanksuq-Nameh) and Aghili (in the Makhzan-al-Advieh) presented some information about Moomiaii and supported its beneficial properties in some diseases [3]. In the tenth century, Ahwazi in the book of Kamel al-Sana’e suggested the Moomiaii as a beneficial compound for cold headache, hemoptysis, asthma and withdrawal of the dead fetus. Also, Avicenna, a famous Persian physician, in his famous book, Qanoon has suggested the Moomiaii as a very effective compound in brain tonic, fertility and many other diseases. In the 12th century, a Persian book called Zakhire Khwarazmshahi was written by Jorjani, and prescribed the Moomiaii for inflammation, ulcers, urinary problems, and prostates [7]. In the TPM approach, medieval Persian physicians introduced Mizaj (dual quality) based on humoral pathology. In this approach, every foods and medicine have own specific nature and make an active (hotness/coldness) and passive (wetness/dryness) effects after consumption in the body [64]. Based on this approach Moomiaii has a hot-dry nature with dissolvent properties and prescribed in different ways (oral, external, and enema) for treatment of diseases related to different body organs [65]. Moomiaii is also known as a solvent of cold-causing compounds, tonic, exhilarating aphrodisiac, ablative of body moistures, and also as a remedy for tremor and facial palsy [66]. Moomiaii accelerates the process and period of wound cleaning and healing from necrotic tissue, granulation, and epithelization via its bacteriostatic activities [67]. Also, Moomiaii as a traditional medicine for the treatment of gastric ulcer and digestive tract disorders may be beneficial due to anti-oxidative, anti-stress, anti-inflammatory and anti-acid properties, but more investigations are needed for approval of clinical applying. In addition, treatment with Moomiaii leads to the proliferation of lymphocytes of cortical thymus layer and significant migration into thymus-dependent zones of lymph nodes and spleen [12]. Prescription of Moomiaii can activate macrophage cell migration in the epithelioid granulomas in struck pulmonary tissue with experimental tuberculosis and cause the transformation of epitheliocellular granulomas to macrophage. As well, treatment with Moomiaii stimulated regeneration of capillaries in the inflammation areas and increased infiltration and absorption of necrotic lesions, and also promoted passage of antibacterial drugs toward inflammation area [68]. Administration of processed shilajit with effective immuno-potentiating properties for 22 HIV patients during 6 months, showed apparent amelioration in the symptoms and augmentation of CD4 and CD8 cell counts [45]. In addition, the processed Moomiaii at a dose of 100 mg twice a day for 90 days to 28 male subjects showed significant decrease in fasting blood glucose and creatinine levels without significant opposing effects on renal profile parameters, including urea, albumin, total protein, globulin, uric acid, bilirubin, alkaline phosphatase, alanine aminotransferase (ALT) or aspartate aminotransferase (AST) [16]. The effects of processed shilajit (250 mg twice/day) on 43 healthy human volunteers for 90 days findings didn’t show significant alterations in kidney or liver function tests but decreased fasting blood sugar, uric acid, and erythrocyte sedimentation rate [16]. Also, Atashbar *et al*. showed that Mumijo can protect against liver damage caused by the consumption of high doses of acetaminophen through reducing the level of main liver enzymes such as ALT, AST, gamma glutamine transferase (GGT), malondialdehyde (MDA), NO, protein carbonyl (PC) and increased the level of glutathione (GPX) after treatment with high doses of acetaminophen in Wistar rats [69]. Furthermore, Khaksari *et al*. revealed that the treatment of brain traumatic rats with Shilajit improved the neurologic outcomes of this situation and reduced brain edema, disruption of the blood-brain barrier (BBB), and intracranial pressure (ICP) after the traumatic brain injury (TBI) [70]. According to several studies’ results, cachexia is the main cause of death in cancer and chemotherapy toxicity may increase the rate of mortality in cancerous patients [71]. Cancer-related cachexia is characterized by loss of skeletal muscle and later progressive functional impairment. Although the significant effect of cachexia on quality of life, chemotherapy toxicity, physical function, and mortality are well recognized, the proper clinical intervention and prevention remain unclear yet. Moomiaii has anabolic effects and can accelerate processes of protein and nucleic acid synthesis, stimulate the energy-providing reactions and can promote transportation of minerals mainly calcium, magnesium, and phosphorus into muscle and bone tissues [12]. Moreover, Moomiaii and its main constituents may affect cancer disease via immunomodulatory activities by improving the immune system through cytokine production, activation of immunological cells and rising the antibody titer in the plasma [32]. Aghili Khorasani in the 16th century wrote a book named al-Adawiyah, which is the most famous book on pharmacology in Persian medicine, mentioned the treatment of various types of tumors, both benign and malignant, by Moomiaii [72]. The treatment of tumor-bearing animals by HA stimulates the murine peritoneal macrophages and neutrophils and also activates splenocytes and increases the secretion of IL-2 at the initial and later stage of the tumor growth [73]. Another study had demonstrated that the treatment of Huh-7 cells with different concentrations of Moomiaii induced apoptosis and inhibited the proliferation of cancerous cells. As well, Moomiaii enhanced miRNA-22 expression level which resulted in significant inhibition of cell proliferation by targeting the c-myc gene and also suppressed the expression level of miRNA-21 [74]. Although several *in-vitro* and *in-vivo* investigations confirmed the beneficial effects of Moomiaii in different chronic and acute diseases, due to probable fungal contamination and existence of some heavy metals in this compound, the recommendation of Moomiaii as an effective natural remedy must be considered with precaution.

## Conclusion

In recent years, numerous *in-vitro*/ *in-vivo* and human studies have been performed regarding the mechanisms of action and medicinal and pharmacological properties of Moomiaii as an ancient medicine and natural remedy. In this review, we discussed the therapeutic effects of Moomiaii with focusing on anti-ulcerogenic, immunomodulatory, antidiabetic, antioxidative and anticancer properties by compiling the data from earlier and recent findings and claims in the TM. Studies in both animals and humans indicated that Moomiaii has a wide range of safety and is free of detrimental effects at the commonly used doses. Moomiaii and its main constituents, HA and FA, known as an inexpensive and safe dietary supplement, mostly prescribed orally and externally in TM with the aim of prevention and treatment of various diseases like genitourinary disorders, jaundice, digestive disorders, diabetes, cancer, nervous disorder, anemia [10]. Although there are various claims in ancient medicine and TM about the effects of Moomiaii, there is not enough scientific support for its using in different diseases. Some recent studies have validated the health beneficial effects of Moomiaii and proved the existing claims regarding their therapeutic activity [75]. However, further *in-vitro*/ *in-vivo* investigations and clinical trials are needed to determine the doses and duration of administration also to confirm the therapeutic effects of Moomiaii for using at the clinical level.

## Acknowledgement

 The financial support of the Tabriz University of Medical Sciences is gratefully acknowledged. The results of this article are derived from the PhD thesis of Solmaz Rahmani Barouji registered in Tabriz University of Medical Sciences, Tabriz, Iran.

## Conﬂict of Interest

 The authors declare that there are no conﬂicts of interest.
